# What to eat for a healthier China?

**DOI:** 10.1016/j.lanwpc.2022.100525

**Published:** 2022-06-27

**Authors:** The Lancet Regional Health – Western Pacific

Food has a central role in Chinese culture. With rapid economic growth and urbanisation, the Chinese dietary pattern has changed considerably, from a predominantly plant-based diet to one including large amounts of animal products, with less intake of cereals and vegetables and more consumption of red meat, processed meat, and sugar-sweetened beverages. In the *Lancet Live Dietary Pattern and Health in China* webinar held on June 8, 2022, *The Lancet Regional Health – Western Pacific* editors and several researchers discussed different dietary patterns and their health and environmental impacts in China.

The transition of dietary patterns in China has been accompanied by a rise in burdens of non-communicable diseases. According to the Global Burden of Diseases, Injuries, and Risk Factors 2017 Diet study, China has the highest age-standardised rates of diet-related cardiovascular disease deaths and cancer deaths worldwide. The latest Chinese Dietary Guidelines released in 2016 recommend eating less red meat and reducing salt, oil, and sugar consumption. Access to a healthy diet relies on individuals' food choices and the availability and affordability of healthy foods. Disparities in dietary habits and food accessibility exist between urban and rural areas, and between different ethnic groups in China. The findings of the China Multi-Ethnic Cohort Study led by Xing Zhao from Sichuan University, suggest that minority ethnic groups (Tibetan, Yi, Miao, Bai, Bouyei, and Dong) who live in Yunnan–Guizhou Plateau and Qinghai–Tibet Plateau consume more salt and eat less whole-grain and fruit compared with those in the Han majority population living in Sichuan Basin, who have relatively healthier and more balanced dietary patterns. This discrepancy in dietary habits contributes to the substantial increase in cardiometabolic risks, especially hypertension, in these minority ethnic groups. The authors of the China Multi-Ethnic Cohort Study suggested that a healthy diet such as the Dietary Approaches to Stop Hypertension could help reduce cardiometabolic risk in the minority ethnic population. One possible next step for this study is to have localised and culturally acceptable nutritional guidance to reduce the dietary-related health risk in those areas.

Food does not just foster our health, but is also a key contributor to greenhouse gas emissions, air and water pollution, and land degradation. Despite health challenges, the change in dietary patterns, especially the substantial increase in red meat consumption, is intensifying environmental problems in China. The shift towards meat-intensive diets between 1980 and 2010 has contributed to around 20% of the total PM_2.5_ pollution increase in China, which results in an estimated 75 000 premature deaths each year. Anna Zhu and colleagues found that plant-based dietary patterns help improve cognitive function caused by long-term PM_2.5_ exposure in older Chinese adults, adding another dimension to understanding the interaction between food, health, and the environment.

The Chinese Government has released several national plans, including the Healthy China 2030 Blueprint, Healthy China Initiative (2019–30), and National Nutrition Plan (2017–30), to promote a healthy and balanced diet on a national level. Understanding the dietary patterns of different populations and barriers to healthy eating is the first step in translating these national policies into practice. However, healthy foods can be expensive. Despite promoting healthy eating by raising individuals’ health awareness and knowledge, the government needs to work with different sectors to develop policies, programmes, and projects to make nutritious foods accessible and affordable to everyone. Furthermore, the impact of food systems on the environment also needs to be considered for future national health and nutrition programmes. As the links between dietary patterns, health, and environmental issues are becoming more evident, promoting a healthy diet is a tangible solution to address both health and environmental challenges in China concomitantly.

